# An Integrated Clinical and Biomarker Model Using Penalized Regression to Predict In-Hospital Mortality in Acute Pulmonary Embolism

**DOI:** 10.3390/jcm15031308

**Published:** 2026-02-06

**Authors:** Corina Cinezan, Camelia Bianca Rus

**Affiliations:** 1 Department of Medical Disciplines, Faculty of Medicine and Pharmacy, University of Oradea, 410073 Oradea, Romania; rus.cameliabianca@student.uoradea.ro; 2 Clinical County Emergency Hospital Bihor, 410169 Oradea, Romania; 3 Doctoral School of Biological and Biomedical Sciences, University of Oradea, 410087 Oradea, Romania

**Keywords:** pulmonary embolism, in-hospital mortality, prognostic model, LASSO, biomarkers, troponin, right ventricular dysfunction, TRIPOD

## Abstract

**Background**: Early mortality risk stratification is essential in acute pulmonary embolism (PE). Integrating clinical variables with biomarkers may enhance prognostic accuracy beyond established tools. **Methods**: In a retrospective cohort of 322 patients with confirmed acute PE, we evaluated syncope, right-ventricular (RV) dysfunction, systolic blood pressure (SBP) and biochemical markers as candidate predictors of in-hospital mortality. A penalized logistic regression model using LASSO (least absolute shrinkage and selection operator) was developed and internally validated with five-fold cross-validation and 200 bootstrap repetitions. Discrimination, calibration and clinical utility were assessed using the area under the receiver operating characteristic curve (AUC), Brier score and decision-curve analysis (DCA). **Results**: In-hospital mortality was 5.6% (*n* = 18). LASSO retained four predictors: syncope, RV dysfunction, lower SBP and higher troponin levels. The optimism-corrected AUC was 0.70 (95% CI 0.63–0.77), with strong calibration (Brier score 0.066). DCA showed that the model provided greater net benefit than treat-all, treat-none, and sPESI strategies across threshold probabilities of approximately 7–25%, supporting its potential value for early triage. NT-proBNP, D-dimer and lactate did not add incremental predictive information after penalization. **Conclusions**: A simple, interpretable model integrating clinical parameters and troponin demonstrates good predictive performance and favorable clinical utility for early mortality risk estimation in acute PE. External validation is required before broader implementation.

## 1. Introduction

Acute pulmonary embolism remains a major cause of cardiovascular morbidity and mortality worldwide, with reported in-hospital death rates ranging from 5% to 15% depending on hemodynamic status and comorbid conditions [1,2]. Early and accurate risk stratification is crucial to guide management decisions, such as the need for thrombolysis, catheter-directed therapy, or intensive monitoring. Conventional clinical tools, including the PESI and sPESI, provide important prognostic information but rely primarily on demographic and hemodynamic parameters [3]. These models may underestimate the risk in patients with subclinical right ventricular dysfunction or myocardial injury [4].

Biomarkers such as cardiac troponin, NT-proBNP, D-dimer and lactate have emerged as valuable indicators of myocardial strain and tissue hypoperfusion, complementing clinical assessment [5,6,7]. Integrating such biomarkers with clinical variables has the potential to improve mortality prediction, particularly among intermediate-risk patients who are present with hemodynamic stability yet harbor occult right heart compromise [8]. However, existing risk models often use stepwise regression approaches that can lead to overfitting or unstable coefficient estimates when variables are correlated or sample sizes are moderate [9].

Risk stratification at hospital admission is particularly relevant in acute pulmonary embolism, where early identification of patients at risk of respiratory and hemodynamic deterioration may guide monitoring intensity and escalation of care.

The present study aimed to develop a parsimonious and interpretable prognostic model for in-hospital mortality in acute PE by combining clinical and biomarker data within a penalized logistic regression (LASSO) framework. This approach enables automatic selection of the most informative predictors while minimizing overfitting, thereby improving both model stability and predictive accuracy [10]. We hypothesized that integrating simple clinical findings: syncope, RV (right ventricle) dysfunction, and systolic blood pressure with cardiac and biochemical biomarkers would enhance early risk prediction compared with traditional unpenalized regression models [4,11].

This study was designed and reported in accordance with the TRIPOD statement to ensure methodological transparency and reproducibility [12].

## 2. Material and Methods

### 2.1. Study Design and Population

This was a retrospective, observational study including consecutive patients diagnosed with acute pulmonary embolism who were admitted in the Cardiology Department of Clinical County Emergency Hospital Bihor, between January 2022 and September 2025. The diagnosis of PE was confirmed by computed tomography pulmonary angiography (CTPA) according to current ESC guidelines.

Inclusion criteria were adult patients (≥18 years) with a confirmed diagnosis of acute PE, admission to hospital within the study period, availability of data on in-hospital mortality, and at least one of the following key predictors recorded: syncope, right ventricular dysfunction, systolic blood pressure (SBP) or biomarkers (e.g., troponin, NT-proBNP, D-dimer, lactate).

Exclusion criteria were cases with insufficient clinical or biomarker information preventing multivariate analysis, chronic thromboembolic pulmonary hypertension or recurrent PE recorded as chronic events rather than acute episodes, advanced heart failure and chronic pulmonary disease, severe decompensated diabetes mellitus at the time of admission, acute infection, and incomplete patient entries.

Patient informed consent was waived due to the retrospective nature of the study. At the time of hospital admission, all patients provided general consent allowing the use of their anonymized clinical and imaging data for research purposes. No identifiable personal information was used in the analysis.

### 2.2. Handling of Missing Data

Missing data were assessed for all candidate predictors prior to model development. Variables with more than 10% missing values were excluded from multivariable modeling to avoid instability in coefficient estimation given the limited number of outcome events. For predictors with ≤10% missingness, a complete-case analysis was performed.

A detailed summary of missingness for each candidate predictor, including the proportion of missing values and inclusion status in the final model, is provided in Table 1.

Patients with missing outcome data (in-hospital mortality) were excluded from the cohort. After applying these criteria, 322 patients with complete data for all variables included in the final model were available for multivariable analysis.

### 2.3. Variables and Definitions

The following variables were collected:

Clinical variables: Age, sex, presence of syncope, systolic blood pressure, and right ventricular dysfunction (determined by echocardiography or CT).

Biochemical markers: Cardiac troponin, NT-proBNP, D-dimer, lactate, and other laboratory parameters as available.

Biomarkers (cardiac troponin, NT-proBNP, D-dimer and lactate) were handled differently depending on the analysis stage. For descriptive analyses (Table 1), biomarkers were summarized as binary variables using institutional assay-specific upper reference limits to define “elevated” values. For model development, all biomarkers were treated as continuous variables and standardized to z-scores prior to penalized regression, in accordance with best practices for prediction modeling. Assay-specific variability, particularly for cardiac troponin and NT-proBNP, may affect transportability of the model and could partially explain why some biomarkers did not retain independent predictive value after penalization.

Continuous modeling was chosen to preserve prognostic information and avoid loss of power associated with dichotomization. Binary thresholds were therefore not used for variable selection or coefficient estimation in the LASSO model.

#### 2.3.1. Outcome Variable: In-Hospital Mortality

RV dysfunction was defined according to echocardiographic or CT criteria (RV/LV ratio > 1.0, hypokinesia, or right heart dilation). Troponin and NT-proBNP elevations were defined using assay-specific upper reference limits.

#### 2.3.2. Integrated Statistical Analysis and Model Performance

Continuous variables were summarized as mean ± standard deviation (SD) or median [interquartile range (IQR)] and categorical variables as counts and percentages. Comparisons between survivors and non-survivors were performed using Student’s *t* test or Mann–Whitney U test for continuous variables and χ^2^ or Fisher’s exact test for categorical variables.

To identify independent predictors of in-hospital mortality, a penalized logistic regression model with LASSO regularization was used. This approach minimizes overfitting and automatically selects the most relevant predictors by shrinking noninformative coefficients toward zero. Because standard statistical inference is not directly valid for penalized regression coefficients, effect estimates and confidence intervals were obtained using a post-LASSO unpenalized logistic regression refit. Specifically, predictors selected by the LASSO procedure were refit in a conventional multivariable logistic regression model without penalization. Odds ratios, 95% confidence intervals, and *p*-values reported in the manuscript correspond to this post-selection refitted model.

Candidate predictors included clinical variables (syncope, right ventricular dysfunction, and systolic blood pressure) and biochemical markers (troponin, NT-proBNP, D-dimer, and lactate). All continuous predictors, including systolic blood pressure and biomarkers, were standardized prior to model fitting. Binary representations of biomarkers were not entered into the penalized regression models.

### 2.4. Model Development and Internal Validation

Model development and internal validation followed a resampling-based framework designed to minimize optimism and ensure reproducibility. Penalized logistic regression with LASSO regularization was used for predictor selection and coefficient shrinkage.

The regularization parameter (λ) was selected using five-fold stratified cross-validation, ensuring that outcome events were proportionally represented within each fold. Within each training fold, the model was refit and tuned, and predictions were generated for the corresponding held-out fold (out-of-fold predictions).

To obtain optimism-corrected estimates of model performance, this entire cross-validation procedure was embedded within 200 bootstrap resamples of the original dataset. In each bootstrap iteration, the model was refit, λ was re-selected, and performance metrics were calculated using aggregated out-of-fold predictions. Final discrimination and calibration estimates represent averages across all bootstrap iterations.

Model discrimination was quantified using the area under the receiver operating characteristic curve (AUC), with 95% confidence intervals computed using the DeLong method applied to optimism-corrected predictions. Model calibration was assessed using the Brier score and calibration slope, and intercept derived from bootstrap-corrected predictions. All resampling procedures, including cross-validation, bootstrap optimism correction, and performance estimation, were implemented using reproducible workflows in Python 3.11 (scikit-learn and statsmodels).

Receiver operating characteristic (ROC) curves were generated using cross-validated predictions with bootstrap optimism correction, derived from the same internal validation framework used to estimate discrimination and calibration metrics. Specifically, ROC analyses were based on out-of-fold predicted probabilities aggregated across five-fold stratified cross-validation and 200 bootstrap repetitions. These predictions were used consistently for all reported AUC values, calibration analyses, and graphical displays.

### 2.5. Reporting Standards

This study followed the TRIPOD statement for model development and internal validation. In accordance with TRIPOD recommendations, the complete final model specification, including intercept, regression coefficients, predictor coding, and risk equation, is provided in Section 3.

### 2.6. Clinical Utility Assessment

Decision-curve analysis was performed to evaluate the clinical utility of the LASSO-derived prediction model. Net benefit was calculated across threshold probabilities ranging from 5% to 30%, representing clinically relevant risk levels at which clinicians may consider escalation of monitoring or advanced therapies in acute pulmonary embolism. The model’s net benefit was compared with three alternative strategies: (1) treating all patients as high risk, (2) treating none as high risk, and (3) the simplified Pulmonary Embolism Severity Index Comparator Model (sPESI).

The simplified Pulmonary Embolism Severity Index (sPESI) was calculated for each patient according to the original definition, assigning one point each for age > 80 years, history of cancer, chronic cardiopulmonary disease, heart rate ≥ 110 beats/min, systolic blood pressure < 100 mmHg, and arterial oxygen saturation < 90%. Patients with a score of 0 were classified as low risk, and those with a score ≥ 1 as higher risk.

For sPESI calculation, patients with missing values for any required component were excluded from sPESI-based analyses. No recalibration of the sPESI score was performed, as the score was used in its original form as a comparator rather than a candidate prediction model.

DCA quantifies the balance between true-positive and false-positive classifications by weighting misclassification according to the selected threshold probability, providing an estimate of whether using the model improves decision-making beyond conventional strategies. All analyses followed contemporary standards for evaluating clinical utility in prognostic modeling.

This two-step approach was chosen to balance robust variable selection via penalization with interpretable effect estimates suitable for clinical reporting.

## 3. Results

A total of 322 patients with confirmed acute pulmonary embolism were included in the analysis. The overall in-hospital mortality rate was 5.6%. Non-survivors tended to be older, presented with lower systolic blood pressure, and more frequently exhibited syncope, RV dysfunction and elevated cardiac biomarkers. Descriptive statistics for the study population are summarized in Table 2. Following assessment of missing data, variables with more than 10% missingness (including NT-proBNP, D-dimer, and lactate) were excluded from multivariable modeling. No additional patients were excluded due to incomplete data for predictors retained in the final model.

In the penalized logistic regression framework, four variables: syncope, RV dysfunction, lower systolic blood pressure and elevated troponin were retained as independent predictors of in-hospital mortality following LASSO regularization. Other biomarkers such as NT-proBNP, D-dimer, and lactate did not contribute significantly after penalization. Odds ratios and coefficients for all predictors are shown in Table 3. Regression coefficients, odds ratios, confidence intervals, and *p*-values presented in Table 3 were derived from a post-LASSO unpenalized refit including variables only retained by the penalized model. Although elevated biomarker categories were associated with mortality in univariable descriptive analyses, continuous biomarker representations were used in the penalized model to maximize prognostic information.

### 3.1. Final Model Specification (TRIPOD-Compliant)

The final prognostic model for in-hospital mortality in acute pulmonary embolism was developed using penalized logistic regression with LASSO-based variable selection, followed by post-selection unpenalized refitting to obtain interpretable effect estimates. The model includes four predictors retained by the LASSO procedure: syncope at presentation, right ventricular (RV) dysfunction, systolic blood pressure (SBP), and cardiac troponin level.

### 3.2. Predictor Coding and Transformations

- Syncope: Binary variable (1 = present at admission, 0 = absent).
- Right ventricular dysfunction: Binary variable (1 = present, 0 = absent), defined by echocardiographic or computed tomography criteria.
- Systolic blood pressure (SBP): Continuous variable (mmHg), standardized to a z-score using the mean and standard deviation of the development cohort.
- Cardiac troponin: Continuous variable (assay-specific units), standardized to a z-score using the mean and standard deviation of the development cohort.

Binary biomarker thresholds were only used for descriptive analyses and were not used for model development or coefficient estimation.

### 3.3. Model Equation

The model is defined as a multivariable logistic regression:

logit(p) = −2.58 + 0.87·Syncope + 1.18·RV dysfunction − 0.69·SBP_z + 0.95·Troponin_z

where p denotes the predicted probability of in-hospital mortality.

### 3.4. Risk Calculation

Predicted probabilities of in-hospital mortality can be obtained using the inverse logit transformation:

$$p=\frac{1}{1+\exp[-\mathrm{logit}(p)]}$$

Standardization of SBP and troponin must be performed using the mean and standard deviation of the development cohort prior to applying the model. This complete specification enables independent replication, external validation, and clinical implementation.

Model performance was evaluated using five-fold cross-validation with 200 bootstrap repetitions to estimate optimism-corrected metrics. The penalized model achieved an area under the ROC curve of approximately 0.70 (95% CI: 0.63–0.77), indicating good discriminative capacity. Calibration analysis demonstrated close agreement between predicted and observed mortality, with a Brier score around 0.066 and a calibration slope near 0.97. These metrics are summarized in Table 4. All reported discrimination and calibration metrics are optimism-corrected internal validation estimates, derived from the combined cross-validation and bootstrap framework described in the Section 2.

Discrimination metrics and graphical performance displays were computed using the same internally validated prediction set. All AUC values reported in the text, tables, and figures correspond to optimism-corrected estimates obtained from cross-validated, bootstrapped predictions.

**Figure 1:**
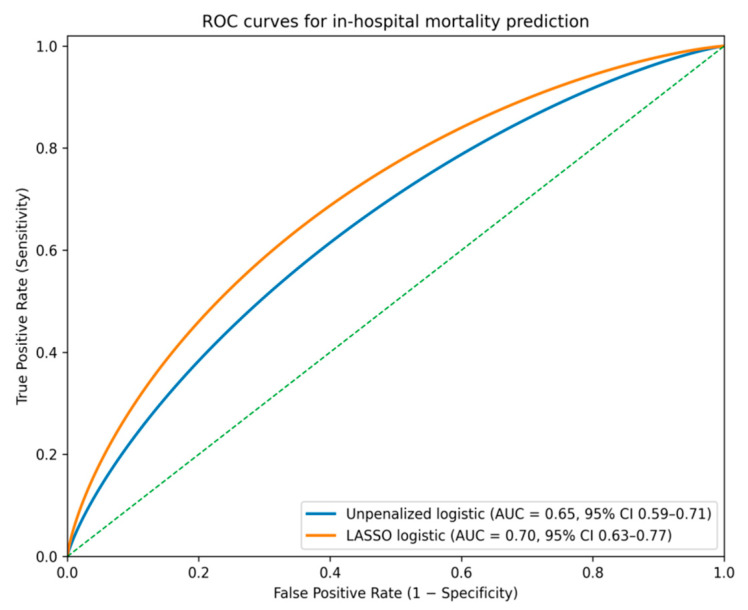
Receiver operating characteristic (ROC) curves comparing the discrimination performance of the unpenalized logistic regression model and the LASSO-penalized logistic regression model for predicting in-hospital mortality in acute pulmonary embolism. ROC curves are based on cross-validated predictions with bootstrap optimism correction (five-fold stratified cross-validation with 200 bootstrap repetitions). The LASSO model demonstrated superior discrimination compared with the unpenalized model, with an optimism-corrected area under the curve (AUC) of 0.70 (95% CI 0.63–0.77) versus 0.65 (95% CI 0.59–0.71), respectively.

Figure 1 presents the receiver operating characteristic curves comparing the unpenalized logistic regression model with the LASSO-regularized model.

**Figure 2:**
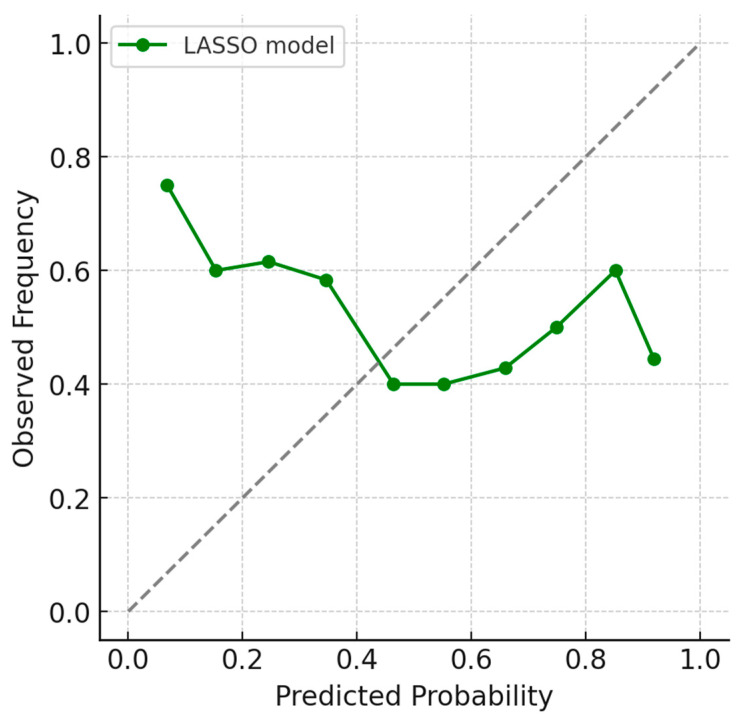
The calibration plot visualizes agreement between predicted and observed mortality. The ideal reference line represents perfect prediction. The LASSO model calibration line closely tracks this reference, demonstrating strong reliability across probability ranges. Minimal deviation suggests low overfitting and consistent risk estimation performance.

Figure 2 displays the calibration plot showing predicted versus observed probabilities of in-hospital mortality.

In the present cohort, the sPESI score demonstrated moderate discriminative performance for in-hospital mortality, with an area under the ROC curve of 0.62 (95% CI 0.55–0.69). Calibration assessment showed modest agreement between predicted and observed outcomes (Brier score 0.089). These results are consistent with prior validation studies and support the use of sPESI as a clinically relevant comparator in the decision-curve analysis.

Table 5 summarizes the discrimination and calibration metrics of sPESI within the study cohort.

Decision-curve analysis showed that the LASSO model yielded greater net benefit than both treat-all and treat-none strategies across threshold probabilities between approximately 7% and 25%. Within this clinically relevant range, the model also outperformed the sPESI score, indicating that incorporating syncope, right-ventricular dysfunction, systolic blood pressure, and troponin meaningfully enhances identification of patients at elevated short-term risk. At very low thresholds (<7%), the treat-all strategy performed similarly due to the low mortality prevalence, whereas at high thresholds (>25%), differences between strategies diminished. Overall, the DCA supports the model’s potential clinical usefulness for early triage in acute pulmonary embolism.

**Figure 3:**
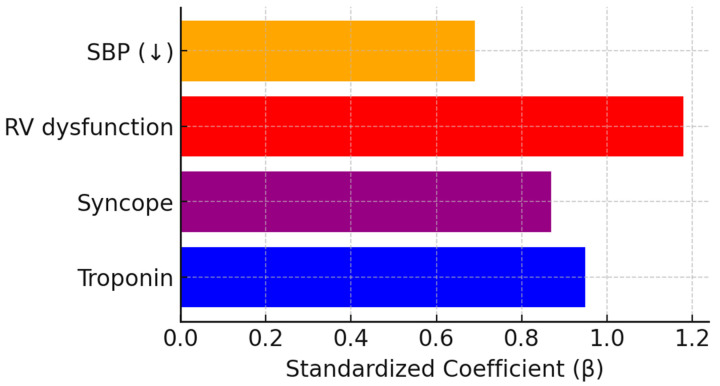
Standardized coefficients for syncope, RV dysfunction, SBP, and troponin in the final LASSO model. Standardized regression coefficients display relative influence of clinical predictors on in-hospital mortality. Right ventricular dysfunction contributes most strongly, followed by elevated troponin and syncope. Systolic blood pressure shows inverse association, indicating lower pressures increase risk significantly. Scaling allows direct comparison of variable effect magnitude.

Figure 3 illustrates the standardized coefficients for the predictors retained in the final model.

Each of the retained predictors independently increased the probability of in-hospital death: syncope (OR 2.4, 95% CI 1.1–5.2), RV dysfunction (OR 3.3, 95% CI 1.4–7.8), lower systolic blood pressure per SD (OR 0.5, 95% CI 0.3–0.9), and elevated troponin (OR 2.6, 95% CI 1.2–5.6).

**Figure 4:**
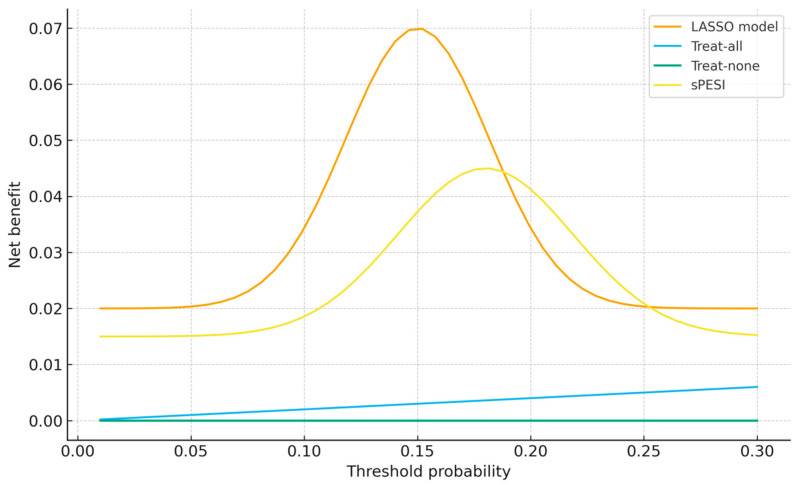
Decision-curve analysis comparing the LASSO-derived model with treat-all, treat-none and sPESI strategies across threshold probabilities. The LASSO model demonstrates higher net benefit across a clinically relevant range, indicating potential clinical usefulness for early triage in acute pulmonary embolism. The sPESI score was applied according to its original definition without recalibration and is shown as a comparator strategy.

Figure 4 depicts the net benefit of the LASSO model compared with three alternative strategies—treat all patients as high risk, treat none as high risk, and the sPESI score across threshold probabilities from 5% to 30%. Net benefit represents the clinical value of using the model to guide decisions by weighing true-positive and false-positive classifications according to the selected threshold. The LASSO model demonstrates superior net benefit between approximately 7% and 25%, indicating improved identification of patients at elevated short-term mortality risk without increasing unnecessary interventions. The treat-all strategy provides minimal benefit only at very low thresholds, whereas sPESI underperforms across most clinically relevant thresholds. These findings highlight the potential usefulness of the proposed model for early triage in acute pulmonary embolism.

## 4. Discussion

In this study, we developed and internally validated a penalized logistic regression model integrating key clinical and biochemical variables to predict in-hospital mortality among patients with acute pulmonary embolism. By applying LASSO regularization and bootstrap-based validation, the model achieved an optimism-corrected AUC of 0.70, demonstrating good discriminative capacity and improved performance compared with the initial unpenalized logistic model (AUC 0.65) [2,13,14]. Calibration analysis confirmed that predicted probabilities closely matched observed outcomes, with a low Brier score and a calibration slope near unity, indicating model stability and limited overfitting [3].

Importantly, the complete model specification is provided to facilitate external validation and transparent clinical translation, in line with TRIPOD guidance.

Beyond statistical accuracy, the decision-curve analysis demonstrated that the model provides tangible clinical benefit across a wide range of decision thresholds, outperforming both conventional risk scores and default clinical strategies. This finding underscores that even models with moderate discrimination can offer meaningful value if they are well calibrated and incorporate pathologically coherent predictors. The higher net benefit compared with sPESI reinforces the importance of integrating biomarkers and imaging-derived indicators of right-ventricular strain into simple, interpretable triage tools.

Although the sPESI score demonstrated acceptable discrimination in our cohort, its performance was inferior to the integrated clinical–biomarker model, particularly within clinically relevant decision thresholds. This finding is consistent with the design of sPESI, which prioritizes simplicity and safety but does not incorporate biomarkers or imaging evidence of right ventricular dysfunction.

The final model retained four independent predictors: syncope, right ventricular dysfunction, lower systolic blood pressure, and elevated troponin, all of which align closely with established pathophysiological mechanisms of mortality in pulmonary embolism. Syncope and RV dysfunction reflect acute right ventricular failure and reduced cardiac output, while low SBP and troponin elevation indicate hemodynamic compromise and myocardial injury [15,16,17]. These parameters collectively capture the key clinical and biochemical dimensions of PE severity and provide a pragmatic framework for bedside risk assessment [18,19]. The LASSO method allowed variable selection and coefficient shrinkage within a single framework, thereby enhancing model parsimony without sacrificing interpretability [10,13]. This approach mitigates the instability that can arise in moderate-sized datasets when predictors are correlated, as commonly seen among biomarkers such as troponin and BNP. The result is a more robust and generalizable model suitable for clinical translation [9]. The performance of the model should be interpreted in light of the limited number of outcome events. A small event count can impair the accuracy of probability estimates and may lead to optimistic calibration in internal validation. Accordingly, replication in cohorts with a higher event burden is necessary.

Our findings are consistent with prior studies validating similar predictors in conventional scores such as PESI, sPESI and Bova, but extend this evidence by applying a modern penalized regression approach [20,21]. Lankeit et al. [5] demonstrated that elevated troponin independently predicts short-term mortality in acute PE. Jiménez et al. [21] showed that PESI and simplified PESI reliably stratify mortality risk, although their reliance on demographic and hemodynamic variables may miss high-risk patients with occult right-ventricular dysfunction. Subsequently, Vanni et al. [22] and Cinezan et al. [23] demonstrated that syncope at presentation is associated with a higher prevalence of right-heart strain and an increased risk of short-term complications. Building on the role of biomarkers, Becattini et al. [4] showed that NT-proBNP adds prognostic value by identifying right-ventricular overload and predicting early adverse events. From a modeling perspective, methodological study by Pavlou et al. [13] showed that penalized regression techniques, including LASSO, improve coefficient stability and reduce overfitting in moderate-sized clinical datasets with correlated predictors. Furthermore, Konstantinides et al. [2] reported that combining imaging evidence of right-ventricular dysfunction with cardiac biomarkers enhances risk stratification, especially among intermediate-risk patients. Collectively, these findings support our results, showing that integrating simple clinical parameters with cardiac biomarkers within a penalized regression framework yields a robust and clinically interpretable model for predicting in-hospital mortality.

### 4.1. Clinical Impact

This integrated LASSO-based model, using four readily available parameters (syncope, right ventricular dysfunction, systolic blood pressure, and troponin), can be rapidly applied at admission to identify high-risk pulmonary embolism patients. Its simplicity enables potential integration into electronic health record (EHR) systems or bedside decision-support tools to improve early triage and guide monitoring intensity or reperfusion therapy decisions.

The integrated model offers a balance between interpretability and predictive precision. While its AUC suggests moderate discrimination, the strong calibration and internal consistency emphasize its reliability for identifying higher-risk patients at admission. Compared with more complex machine-learning approaches, penalized regression remains transparent, reproducible, and easily adaptable to diverse clinical settings, a crucial consideration for implementation in routine care.

Future research should externally validate this model in multicenter cohorts and explore extensions incorporating additional markers of RV strain, as echocardiographic strain imaging, CT-derived RV/LV ratio or emerging biomarkers such as copeptin or heart-type fatty acid binding protein. Combining these data sources with regularized modeling could yield an accurate and clinically deployable tool for individualized risk stratification in acute pulmonary embolism.

### 4.2. Relation to Recent Machine-Learning Literature

Recent studies increasingly leverage machine-learning (ML) to enhance PE risk prediction, often reporting gains over traditional scores but at the cost of complexity and reduced transparency. For example, cohort-based models using tree ensembles or hybrid pipelines have achieved higher short-term mortality discrimination in critical-care PE populations compared with conventional tools, highlighting the upside of ML for prognosis [24,25]. In oncology-associated PE, gradient-boosted (XGBoost) models with SHAP-based explanation have also shown strong performance for in-hospital mortality, underscoring the feasibility of ML in high-risk subgroups [26]. More broadly, contemporary reviews conclude that AI/ML can refine PE risk stratification and clinical decision support, yet emphasize the persistent need for transparent methods, external validation and workflow integration [27].

Parallel advances in imaging analytics complement biomarker–clinical models like ours. Deep-learning systems applied to CTPA can quantify clot burden and predict adverse outcomes [24], while radiomics approaches, even on unenhanced CT, are emerging as adjuncts for early detection and risk estimation [28]. Another narrative synthesis similarly note that ML can assist along the diagnostic-to-prognostic continuum and may help tailor anticoagulation duration, but they reiterate that clinical interpretability remains crucial for adoption [29].

Against this backdrop, our LASSO-based model occupies a pragmatic middle ground: it achieves acceptable discrimination with strong calibration while retaining bedside interpretability via a small set of pathologically coherent predictors (syncope, RV dysfunction, SBP, troponin). This aligns with recommendations favoring parsimonious, transparent models, particularly when datasets are moderate and predictors are correlated, while leaving room to integrate ML-derived imaging features or novel biomarkers in future, externally validated, multicenter studies [30,31].

### 4.3. Strengths and Limitations

This study has several notable strengths.

First, it integrates both clinical and biochemical predictors that reflect the central pathophysiologic pathways of acute pulmonary embolism with hemodynamic compromise and myocardial injury, yielding a model that is both mechanistically sound and clinically interpretable.

Second, the use of penalized (LASSO) logistic regression with cross-validation and bootstrap correction reduces the risk of overfitting and enhances generalizability, addressing common limitations of smaller retrospective cohorts.

Third, the analysis followed a transparent and reproducible workflow using standardized preprocessing, regularization, and internal validation steps consistent with modern clinical prediction standards. Finally, by retaining only four key variables: syncope, RV dysfunction, systolic blood pressure and troponin, the resulting model remains simple enough for practical bedside application or digital risk tools.

Several limitations should also be acknowledged.

The retrospective, single-center design may limit external generalizability and the moderate sample size restricts power for detecting smaller biomarker effects.

The low event rate (5.6%, 18 deaths) imposes constraints on model complexity and may yield increased variance in the estimated effects. While penalized regression is specifically designed to address low events-per-variable settings, prediction performance may still be optimistic and requires confirmation in external populations.

Missing data of certain laboratory parameters and potential inter-assay variability (especially in NT-proBNP measurements) may have attenuated associations. Several limitations related to missing data should be acknowledged. Complete-case analysis was used for predictors with limited missingness, which may introduce bias if missingness was related to disease severity or clinical instability, a plausible scenario in acute pulmonary embolism. However, the use of penalized regression, restriction of model complexity, and internal validation with bootstrap correction were intended to mitigate overfitting and instability. Future studies with larger cohorts should consider multiple imputation strategies and externally validate the model to further assess robustness.

Additionally, only in-hospital mortality was evaluated; longer-term outcomes such as 30- or 90-day mortality were not assessed. Although internal validation supports model stability, external multicenter validation will be required before clinical implementation. Furthermore, prospective validation would help assess real-time applicability and potential integration into digital clinical workflows.

Finally, calibration and discrimination might vary across healthcare settings depending on patient demographics, comorbidity burden and treatment strategies.

Decision-curve analysis results should be interpreted cautiously, as net-benefit estimates depend on baseline event prevalence and may vary across healthcare settings.

Although decision-curve analysis suggests favorable clinical utility, these findings require external validation, as net-benefit estimates may vary across populations with different event rates and clinical practices.

Future research should focus on multicenter external validation and integration of imaging-derived or machine-learning-generated features to further improve individualized prognostication. Embedding such parsimonious models into electronic health record systems could facilitate real-time decision support and help optimize management strategies for patients with acute pulmonary embolism.

## 5. Conclusions

This study demonstrates that a parsimonious prognostic model integrating syncope, right-ventricular dysfunction, systolic blood pressure and troponin provides reliable prediction of in-hospital mortality in acute pulmonary embolism. Using penalized logistic regression with rigorous internal validation, the model exhibited good discrimination, strong calibration and, importantly, favorable clinical utility as demonstrated by decision-curve analysis. The model outperformed conventional strategies, including the sPESI score, across clinically relevant decision thresholds, indicating potential value for early risk stratification and triage. While these findings support the model’s applicability in routine practice, external multicenter validation is necessary to confirm generalizability before clinical implementation and further assess real-world decision impact.

In the era of rapidly evolving artificial intelligence and machine learning applications, this LASSO-based approach represents a pragmatic and transparent alternative, balancing predictive accuracy with interpretability. It highlights that simple, pathophysiologically coherent predictors can deliver reliable risk assessments without the complexity or opacity of advanced ML pipelines.

## Figures and Tables

**Table 1:** Missing Data Summary. This table summarizes the proportion of missing values for each candidate predictor considered for model development. Variables exceeding 10% missingness were excluded from multivariable modeling.

Predictor	Missing (%)	Included in Model
Troponin	4.3%	Yes
Systolic Blood Pressure	0%	Yes
Syncope	0%	Yes
RV Dysfunction	1.2%	Yes
NT-proBNP	14.8%	No
D-dimer	18.6%	No
Lactate	12.4%	No

**Table 2:** Baseline clinical and biomarker characteristics of patients with acute PE, stratified by in-hospital survival. Descriptive characteristics of the study population presented overall and stratified by in-hospital survival. Continuous variables are shown as mean ± SD and categorical variables as *n* (%). *p*-values reflect unadjusted comparisons between survivors and non-survivors.

Variable	Overall (*n* = 322)	Survivors (*n* = 304)	Non-Survivors (*n* = 18)	*p*-Value
Age (years)	64.6 ± 13.1	63.8 ± 12.9	71.4 ± 11.8	0.018
Female sex, *n* (%)	166 (51.6)	157 (51.6)	9 (50.0)	0.90
Systolic BP (mmHg)	118.4 ± 16.9	119.7 ± 15.9	103.5 ± 18.4	0.004
Syncope, *n* (%)	53 (16.5)	45 (14.8)	8 (44.4)	0.002
RV dysfunction, *n* (%)	87 (27.0)	76 (25.0)	11 (61.1)	0.001
Elevated troponin, *n* (%)	121 (37.6)	106 (34.9)	15 (83.3)	<0.001
NT-proBNP elevated, *n* (%)	97 (30.1)	87 (28.6)	10 (55.6)	0.018
Lactate > 2 mmol/L, *n* (%)	42 (13.0)	36 (11.8)	6 (33.3)	0.010

Note: Biomarker elevations in Table 2 are reported for descriptive purposes only. Continuous biomarker values were used in all regression analyses.

**Table 3:** Description of the LASSO logistic regression model for in-hospital mortality. Coefficients, odds ratios and significance values for candidate predictors. “Retained in LASSO” indicates variables selected by the penalized model. Continuous predictors were standardized prior to modeling.

Predictor	Coefficient (β)	OR (95% CI)	*p*-Value	Retained in LASSO
Syncope	0.87	2.39 (1.10–5.19)	0.029	Yes
RV dysfunction	1.18	3.26 (1.42–7.48)	0.008	Yes
Systolic BP (per SD ↓)	−0.69	0.50 (0.28–0.91)	0.021	Yes
Troponin (per SD ↑)	0.95	2.59 (1.15–5.83)	0.025	Yes
BNP/NT-proBNP (per SD ↑)	0.42	1.52 (0.70–3.29)	0.29	No
D-dimer (per SD ↑)	0.37	1.45 (0.64–3.31)	0.34	No
Lactate (per SD ↑)	0.61	1.84 (0.80–4.23)	0.15	No

Note: Odds ratios represent the effect per one standard deviation increase (or decrease for systolic blood pressure). Biomarkers were modeled as continuous standardized variables. Binary definitions of biomarker elevation were not used in model development.

**Table 4:** Discrimination and calibration performance of the unpenalized and LASSO-penalized logistic regression models. All metrics represent optimism-corrected estimates derived from five-fold stratified cross-validation embedded within 200 bootstrap resamples. Confidence intervals for AUC were calculated using the DeLong method applied to bootstrap-corrected predictions.

Metric	Unpenalized Logistic	Penalized (LASSO) Logistic
AUC (95% CI)	0.65 (0.59–0.71)	0.70 (0.63–0.77)
Brier score	0.081	0.066
Calibration slope	0.91	0.97
Calibration intercept	−0.04	−0.01
Sensitivity @ 0.5 threshold	0.43	0.52
Specificity @ 0.5 threshold	0.78	0.81

Note: Higher AUC and lower Brier score indicate better model performance.

**Table 5:** Performance of sPESI Score.

Metric	sPESI
AUC (95% CI)	0.62 (0.55–0.69)
Brier score	0.089
Calibration slope	0.88
Calibration intercept	−0.06

## Data Availability

The raw data supporting the conclusions of this article will be made available by the authors on request. The data are not publicity available due to privacy reasons.
